# Minimally invasive trocar-assisted reduction of an acute locked multifragmentary posterior fracture-dislocation of the proximal humerus without internal fixation: a case report

**DOI:** 10.1016/j.xrrt.2026.100838

**Published:** 2026-08-05

**Authors:** Thomas Lenssens, Yannick Goubau

**Affiliations:** Department of Orthopaedic Surgery and Traumatology, AZORG Hospital, Aalst, Belgium

## Introduction

Traumatic posterior glenohumeral dislocations are relatively uncommon orthopedic injuries and are frequently overlooked at initial presentation. However, they are associated with a high incidence of concomitant osseous and/or soft tissue injuries. Early recognition and appropriate management of a posterior (fracture-)dislocation of the proximal humerus are essential to limit morbidity. Nevertheless, evidence-based treatment protocols are currently lacking.

This report presents the case of an acute locked and multifragmentary posterior fracture-dislocation of the proximal humerus, which was reduced by minimally invasive means using manipulations with an arthroscopic trocar and subsequently treated without internal fixation.

This case contributes to the limited literature on minimally invasive treatment strategies for acute posterior fracture-dislocations of the proximal humerus by demonstrating the successful use of a trocar-assisted reduction technique in a particularly challenging injury pattern (ie, locked and multifragmentary).

## Clinical presentation

A 43-year-old, right-hand dominant man presented to our emergency department following a left shoulder injury sustained during an accidental bicycle fall the previous evening. The patient had a history of polysubstance abuse.

Physical examination of the shoulder was limited because of pain inhibition. No fixed internal rotation deformity of the left shoulder was observed. Neurovascular examination revealed no abnormalities.

**Figure 1:**
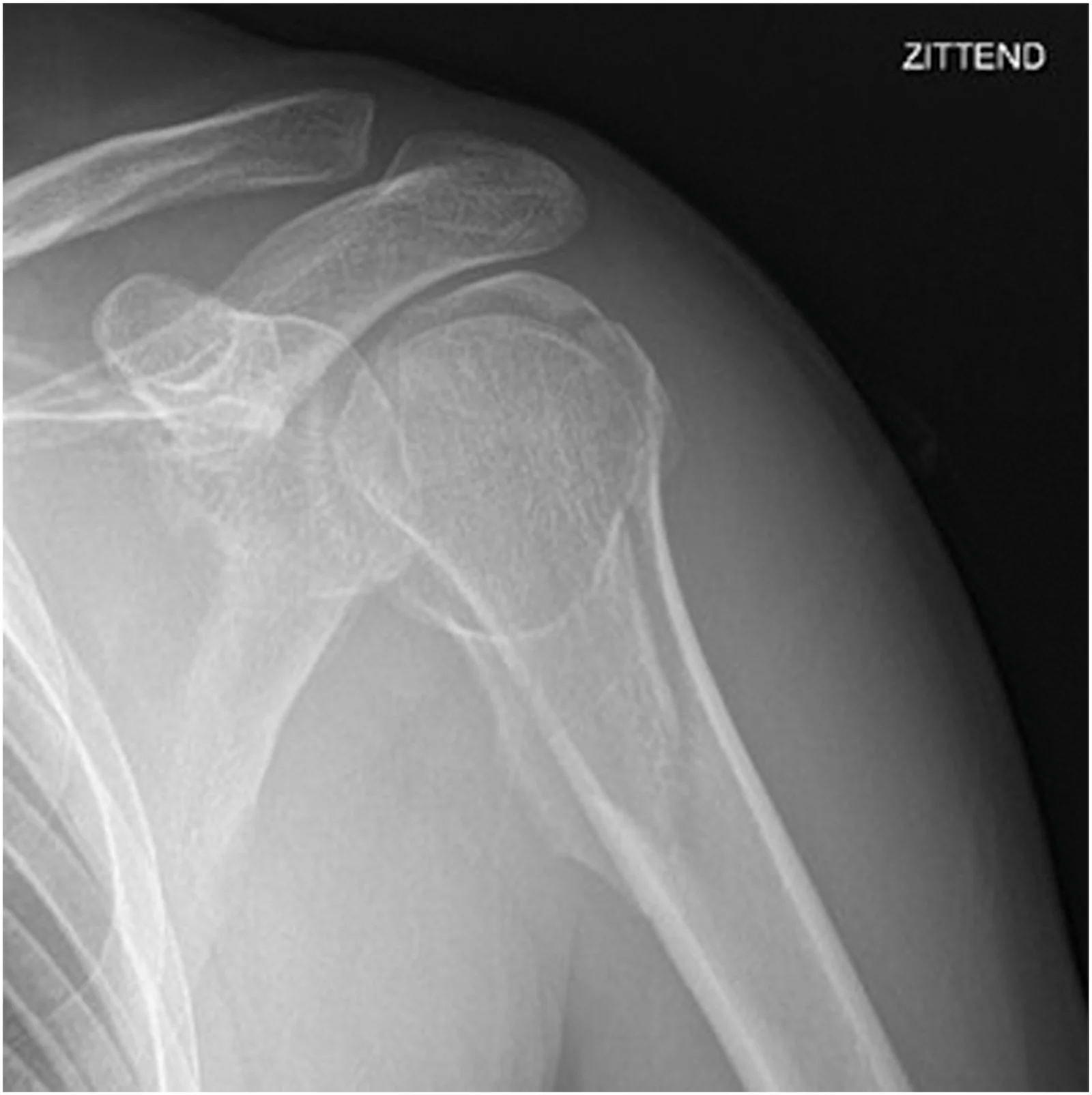
Preoperative conventional anteroposterior radiograph of the left shoulder showing a three-part proximal humeral fracture with a light bulb sign.

Conventional radiographs demonstrated a three-part proximal humeral fracture with a light bulb sign on the anteroposterior view (Fig. 1).

**Figure 2:**
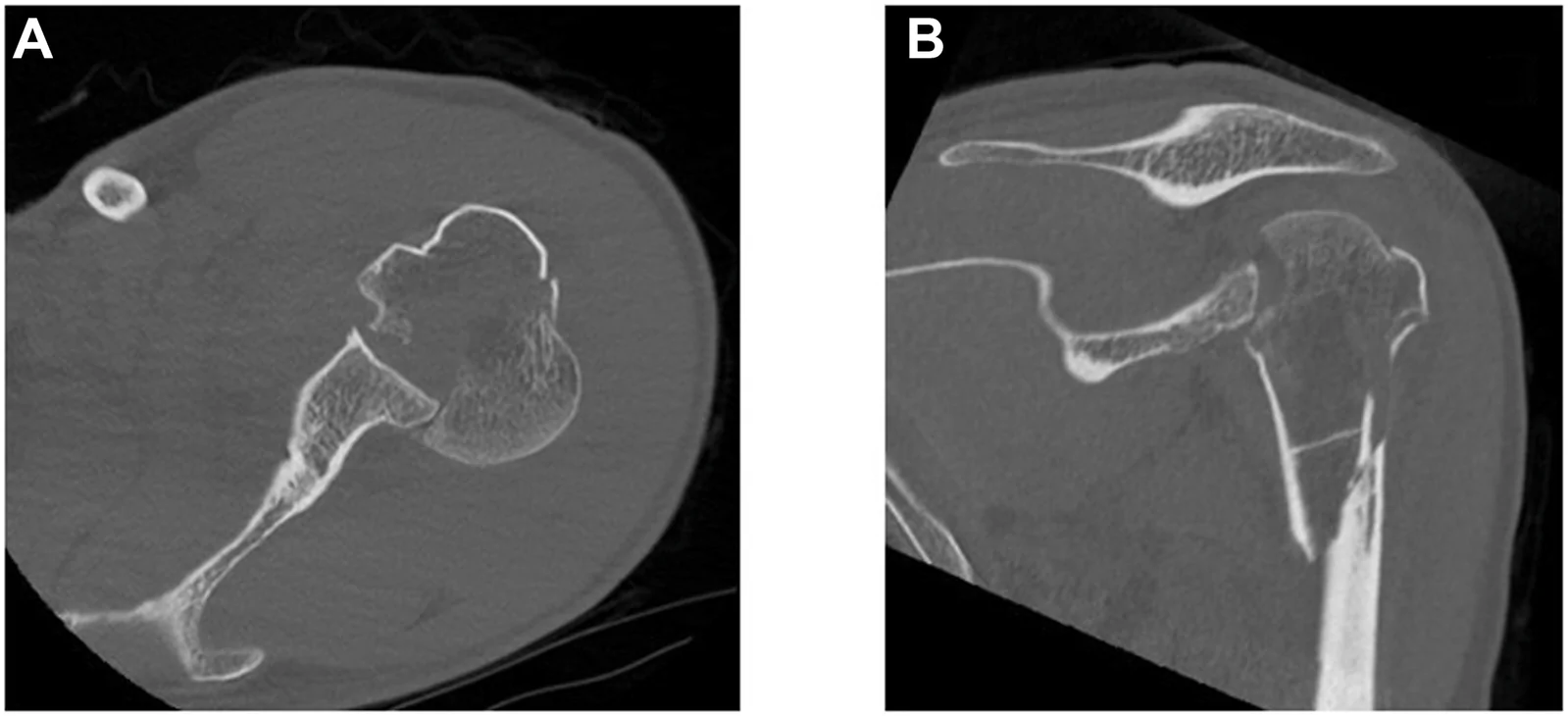
postoperative axial (**A**) and coronal (**B**) CT scan of the left shoulder showing a multifragmentary posterior fracture-dislocation of the proximal humerus. *CT*, computed tomography.

Additional investigation with computed tomography confirmed the diagnosis of a multifragmentary posterior fracture-dislocation of the proximal humerus (Fig. 2), as well as the presence of an engaged large reverse Hill-Sachs lesion, involving approximately 15% of the humeral head articular surface. No head-split component was identified.

## Surgical technique

Reduction under general anesthesia was performed within 48 hours after the initial trauma. The patient was placed in the beach-chair position. The affected upper limb was disinfected and draped. Intraoperative fluoroscopic control was used. Standard closed reduction maneuvers were unsuccessful.

**Figure 3:**
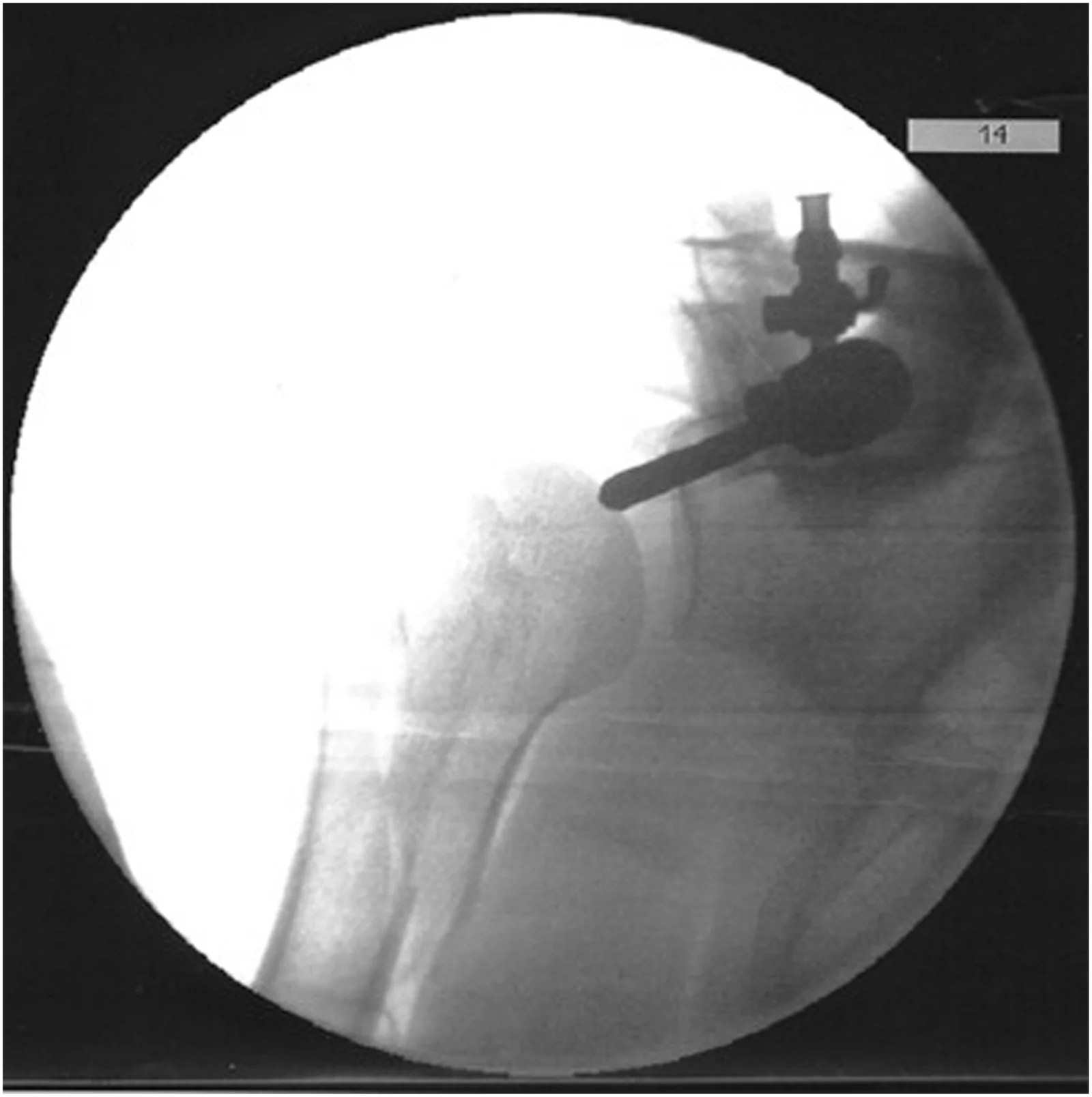
Intraoperative fluoroscopy confirming trocar-assisted glenohumeral reduction of the posterior fracture dislocation of the proximal humerus.

To facilitate reduction, a standard posterior portal incision was made, and a blunt-tip trocar was inserted without use of the arthroscope itself. The humeral head could relatively easily be reduced to its anatomic position using posterior manipulations with the trocar. A control fluoroscopy showed a satisfactory reduction, which was stable in external rotation. However, a recurrent posterior dislocation occurred during internal rotation. Following a repeat reduction maneuver and application of an external rotation splint, reduction could subsequently be maintained (Fig. 3). Therefore, further internal fixation was not performed.

## Follow-up

**Figure 4:**
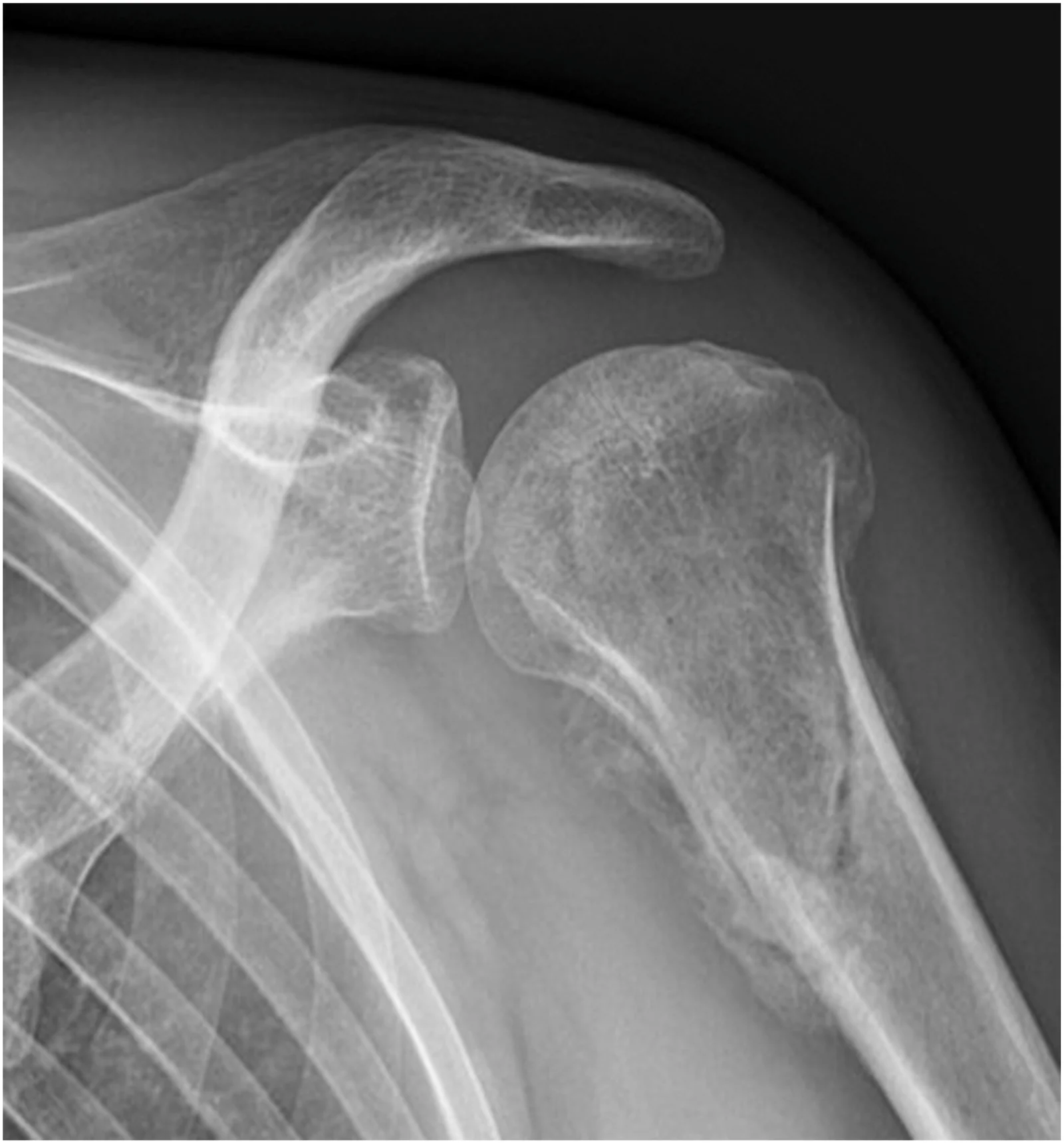
Conventional anteroposterior radiograph at 6 weeks demonstrated fracture union.

The postoperative course was uneventful. The affected upper limb was full-time immobilized in an external rotation splint for 4 weeks. From postoperative weeks 4 to 6, the patient was permitted to remove the splint to perform active external rotation and scapular plane abduction exercises. Fracture union was observed at 6 weeks postoperative (Fig. 4), the splint was therefore discontinued and active as well as passive range-of-motion exercises were progressively advanced. To minimize the risk of recurrent posterior instability, the patient was instructed to avoid the posterior apprehension position (ie, forward flexion combined with internal rotation) during the first 12 post-operative weeks.

**Figure 5:**
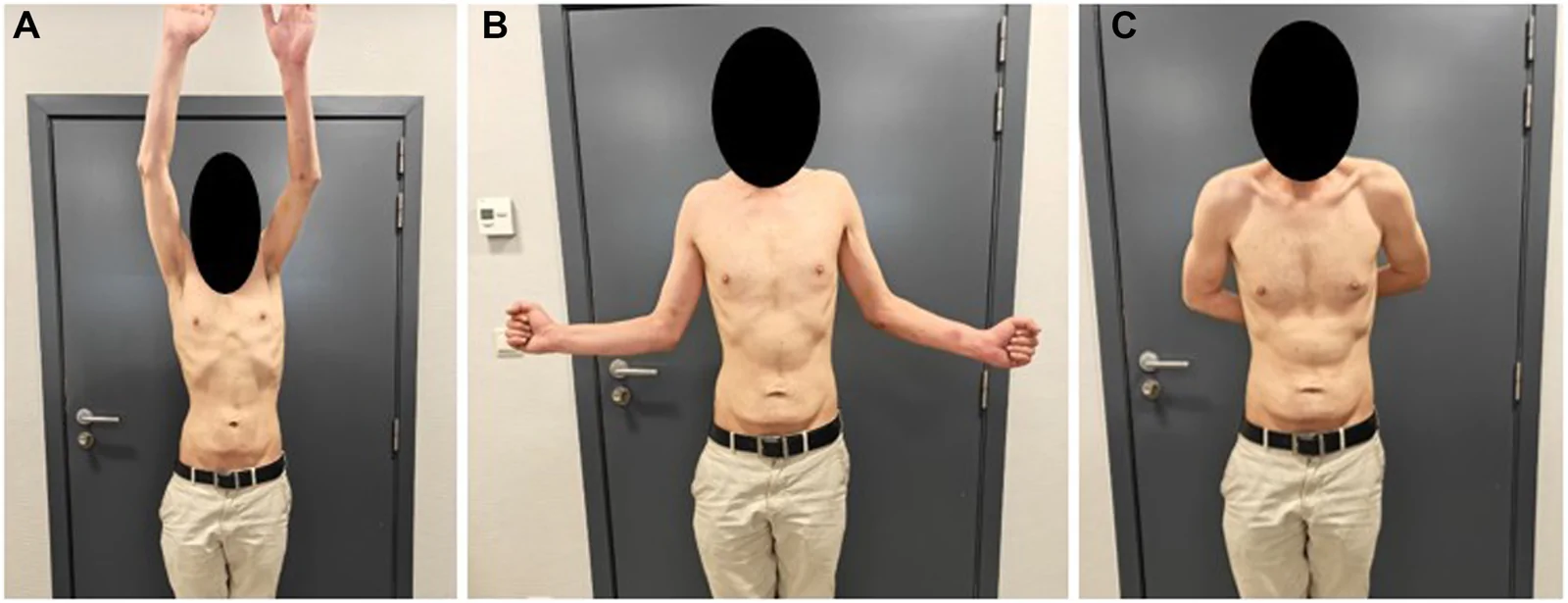
Physical examination at 4-month follow-up show 150° of forward flexion (**A**) with some scapular compensation, 60° of external rotation (**B**), and internal rotation to the lower thoracic region (**C**).

At the last follow-up, 4 months after reduction, the patient reported minimal pain. Physical examination revealed nearly full range of motion of the shoulder, with 150° of forward flexion but with some scapular compensation, 135° of abduction, internal rotation to the lower thoracic region (T12), and 60° of external rotation (Fig. 5).

## Discussion

### Epidemiology

The glenohumeral joint is the most commonly dislocated joint in the human body. While the vast majority of cases involve anterior shoulder dislocations, only 2% to 5% of all glenohumeral dislocations occur posteriorly.^12^

The highest incidence of posterior glenohumeral dislocations was observed in middle-aged men.^8^^,^^10^ These injuries typically occur following high-energy trauma, in which a direct axial force is applied to a shoulder positioned in adduction, forward elevation, and internal rotation. In addition, posterior glenohumeral dislocations can arise from an unbalanced muscle contraction, such as during an epileptic seizure or electrocution.^10^^,^^12^

A posterior dislocation of the proximal humerus rarely occurs as an isolated injury. Up to 65% of cases have associated bony and/or soft-tissue injuries. In a systematic review by Rouleau and Hebert-Davies,^13^ 34% of posterior glenohumeral dislocations were accompanied by 1 or more fractures at the level of the proximal humerus. Fracture lines most frequently involved the anatomic neck of the humerus (55%), followed by the lesser tuberosity (42%) and greater tuberosity (23%). Concomitant fractures of the humeral diaphysis, scapula, or clavicle were rare (6%).

Early diagnosis of a proximal humeral (fracture-)dislocation is essential. Delayed treatment is associated with an increased risk of poor long-term functional outcomes and complications, including avascular necrosis (AVN). Nevertheless, the rate of initially missed diagnoses can reach up to 79%.^10^^,^^12^

### Avascular necrosis

Posttraumatic AVN of the humeral head remains a notorious, although relatively uncommon, complication following a proximal humeral fracture(-dislocation). The precarious vascular anatomy of the humeral head predisposes to AVN after trauma.^7^^,^^9^

The reported incidence of AVN following open reduction and internal fixation with angle-stable implants ranges from 7.9% to 10%.^2^^,^^15^^,^^16^ Neither recent meta-analyses demonstrated significant difference in AVN rates between surgically and conservatively managed proximal humeral fractures^3^^,^^19^ nor do fracture-dislocations appear to independently predispose to its development.^6^^,^^9^

Certain radiographic features have been associated with an increased risk of fracture-induced humeral head ischemia, including a short medial metaphyseal head extension or “calcar segment,” disruption of the medial periosteal hinge, and fractures involving the anatomic neck.^6^ Notably, none of these predictors of ischemia were present in the current case.

However, the development of AVN does not solely depend on the initial postfracture vascular status, as revascularization of the humeral head may still occur over time. Consequently, achieving an anatomic and stable reduction is considered essential to facilitate revascularization.^1^^,^^14^ In addition, early fracture stabilization (ie, within 48 hours after trauma) appears to reduce humeral head ischemic time and may thereby contribute to preservation of humeral head viability.^14^ Moreover, delayed intervention may result in persistent posterior displacement of the humeral head and progressive posterior capsular distension, thereby increasing the difficulty of reduction and the risk of posterior instability.^5^

### Treatment

Given the limited evidence available in the current literature and the pathoanatomical complexity of acute posterior fracture-dislocations of the proximal humerus, no consensus currently exists regarding definitive treatment guidelines. Nevertheless, there is general agreement on several factors that should guide therapeutic decision-making, including the duration of the injury and the patient's age.^8^^,^^10^

A wide variety of therapeutic options have been proposed to address this type of injury,^8^^,^^10^ each associated with specific limitations and potential complications. Humeral head–preserving procedures carry the risk of AVN. In addition, internal fixation with angle-stable plate osteosynthesis may result in implant-related irritation and/or intra-articular screw perforation.^2^^,^^15^^,^^16^ Although arthroplasty may circumvent these complications, functional outcomes remain variable, and this approach is generally not considered the first choice in younger patients,^17^ as in this case.

Based on an extensive review of the literature, Kokkalis et al^8^ proposed a treatment algorithm for posterior fracture-dislocations of the proximal humerus. They recommend open reduction and internal fixation in case of an acute (<3 weeks) injury with a complex fracture pattern, as previously advocated by Robinson and colleagues.^11^

Varghese et al^18^ and Godenèche et al^5^ previously described minimally invasive arthroscopy-assisted reduction methods for acute posterior glenohumeral dislocations associated with an anatomic neck fracture. Arthroscopy-assisted reduction offers the additional advantage of immediate arthroscopic assessment and documentation of any associated osteochondral, capsular, labral, ligamentous, or tendon injury.^5^^,^^18^ Nevertheless, it has also been reported that even three-part posterior fracture-dislocations of the proximal humerus can be successfully managed by closed reduction.^4^ Following successful reduction — resulting in a minimally displaced proximal humeral fracture — a conservative treatment strategy may be adopted, with satisfactory functional and radiographic outcomes reported in the literature.^3^

Despite the risk of secondary fracture displacement, we believe — in agreement with De Wall et al^4^ — that an attempt at closed reduction should always be considered in order to minimize surgical morbidity. A successful closed or minimally invasive trocar- or arthroscopy-assisted reduction may offer a number of potential advantages over the conventional open approach.^4^^,^^5^^,^^18^:

• Preservation of the integrity of the capsuloligamentous structures, which promotes glenohumeral stability and ligamentotaxis and consequently contributes to primary fracture stability.
• Minimization of disruption to the surrounding soft-tissue planes, which may limit subsequent scar tissue formation and thereby facilitate secondary surgical procedures if required.
• Preservation of native bone stock.
• Limitation of additional iatrogenic devascularization.

## Conclusion

We propose that, in cases of acute locked and multifragmentary posterior fracture-dislocations of the proximal humerus in which humeral head viability is not compromised, a minimally invasive trocar-assisted reduction without internal fixation may be considered a valid and safe initial treatment strategy, provided that an early (ie, <48 hours after the trauma) and near-anatomic reduction with intraoperative fracture stability in external rotation can be achieved. Restoration of the medial periosteal hinge appears to be a key factor in ensuring humeral head revascularization. If these conditions cannot be achieved, conversion to open reduction (with internal fixation) should be considered.

## Disclaimers:

Funding: No funding was disclosed by the authors.

Conflicts of interest: The authors, their immediate family, and any research foundation with which they are affiliated have not received any financial payments or other benefits from any commercial entity related to the subject of this article.

Patient consent: Obtained.

## Declaration of generative AI and AI-assisted technologies in the writing process

During the preparation of this work, the authors used ChatGPT (GPT-5.5, from OpenAI) in order to improve readability and language. After using this tool/service, the authors reviewed and edited the content as needed and take full responsibility for the content of the publication.
